# Precision Immuno-Oncology: Prospects of Individualized Immunotherapy for Pancreatic Cancer

**DOI:** 10.3390/cancers10020039

**Published:** 2018-01-30

**Authors:** Jiajia Zhang, Christopher L. Wolfgang, Lei Zheng

**Affiliations:** 1 Departments of Oncology and Surgery, Sidney Kimmel Comprehensive Cancer Center, Johns Hopkins University School of Medicine, Baltimore, MD 21287, USA; zhangjiajia@jhmi.edu (J.Z.); cwolfga2@jhmi.edu (C.L.W.); 2 Bloomberg-Kimmel Institute for Cancer Immunotherapy, Baltimore, MD 21287, USA; 3 Pancreatic Cancer PMCoE Program, Johns Hopkins University School of Medicine, Baltimore, MD 21287, USA

**Keywords:** pancreatic cancer, pancreatic ductal adenocarcinoma, precision medicine, immunotherapy, immune checkpoint, vaccine, tumor microenvironment

## Abstract

Pancreatic cancer, most commonly referring to pancreatic ductal adenocarcinoma (PDAC), remains one of the most deadly diseases, with very few effective therapies available. Emerging as a new modality of modern cancer treatments, immunotherapy has shown promises for various cancer types. Over the past decades, the potential of immunotherapy in eliciting clinical benefits in pancreatic cancer have also been extensively explored. It has been demonstrated in preclinical studies and early phase clinical trials that cancer vaccines were effective in eliciting anti-tumor immune response, but few have led to a significant improvement in survival. Despite the fact that immunotherapy with checkpoint blockade (e.g., anti-cytotoxic T-lymphocyte antigen 4 [CTLA-4] and anti-programmed cell death 1 [PD-1]/PD-L1 antibodies) has shown remarkable and durable responses in various cancer types, the application of checkpoint inhibitors in pancreatic cancer has been disappointing so far. It may, in part, due to the unique tumor microenvironment (TME) of pancreatic cancer, such as existence of excessive stromal matrix and hypovascularity, creating a TME of strong inhibitory signaling circuits and tremendous physical barriers for immune agent infiltration. This informs on the need for combination therapy approaches to engender a potent immune response that can translate to clinical benefits. On the other hand, lack of effective and validated biomarkers to stratify subgroup of patients who can benefit from immunotherapy poses further challenges for the realization of precision immune-oncology. Future studies addressing issues such as TME modulation, biomarker identification and therapeutic combination are warranted. In this review, advances in immunotherapy for pancreatic cancer were discussed and opportunities as well as challenges for personalized immune-oncology were addressed.

## 1. Introduction

Pancreatic cancer is the fourth leading cause of cancer death for both men and women, with an annual incidence of approximately 53,000 new cases in the United States, of whom 43,000 are expected to die [1]. Despite a better understanding of tumor biology and optimization of current treatment modalities, 5-year survival rate of pancreatic cancer is only 5–6% [2]. These sobering results have spawned the efforts spent on developing novel therapies to improve the treatment outcomes. Immunotherapy, which targets cancer cells by augmenting the immune system, has become a game changer in modern cancer cares. Emerging immunotherapeutics including immune checkpoint blockade antibodies and CAR T cell therapies have led to durable response among responsive patients. However, challenges remain as only an objective response rate of 10–30% was observed among those receiving single agent immunotherapy. There is a growing need for individualized medicine solutions to guide patient selection by predicting treatment response, to spare patients from ineffective treatment, and also to avoid toxicity associated with immunotherapy.

With rapid advancement in the technology of next generation sequencing and novel bioinformatics platforms, molecular and genetic profiling of tumors has become an integral venue to guide personalized cancer cares. Opportunities for precision medicine have been expanded to the field of immune-oncology. Integrating immunotherapy with precision medicine by leveraging molecular, genomic, cellular, clinical, behavioral, physiological, and environmental parameters to tailor immunotherapy options has generated enormous interests. PD-L1 status, mutation burden and neoantigen load has been shown in various cancer types to predict positive response to immune checkpoint inhibitors. More challenges lie in validations of the clinical values of these biomarkers in selecting the patients for immunotherapy. Particular opportunities and challenges of personalized immunotherapy exist for pancreatic cancers. 

## 2. Overview of Immune-Biology of Pancreatic Cancer

**Figure 1:**
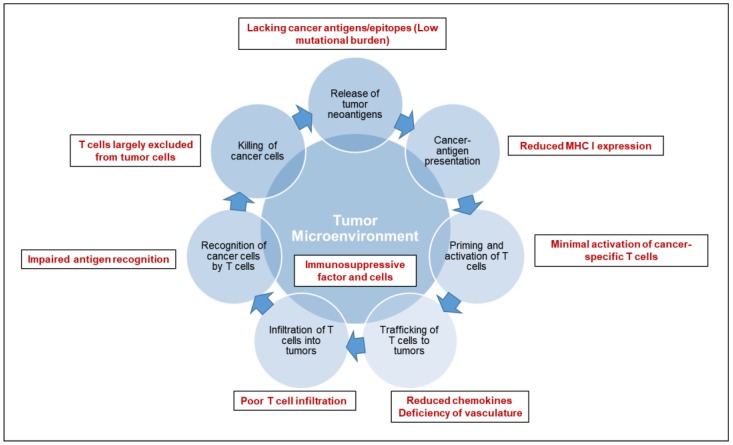
Deficiencies in the tumor microenvironment of pancreatic cancer. The mechanistic processes of anti-tumor immune response are delineated in the diagram. The deficiencies in tumor microenvironment that lead to the failure of anti-tumor immune response are highlighted in red.

Cancer immunotherapy is based on the exquisite specificity of both antibodies and T cells to differentiate the subtle differences between cancer and normal cells and thus mediate a response against tumor cells. To trigger an effective killing of cancer cells, a series of stepwise events must be initiated and allowed to proceed and be expanded iteratively [3,4]: (1) release of tumor specific or associated antigens; (2) antigen presentation (dendritic cells/APCs); (3) priming and activation of T cells; (4) trafficking of T cells to tumors (CTLs); (5) infiltration of T cells into tumors; (6) recognition of cancer cells by T cells; (7) killing of cancer cells. Each step of anti-tumor immune response is characterized by the coordination of numerous factors, with stimulatory factors promoting immunity and inhibitory factors reducing immune activity or keeping the process in check. Therefore, cancer immunotherapy has been attempted by targeting each of the rate-limiting steps. Over the last decade, researches have suggested an immunosuppressive TME (Figure 1) as the fundamental basis for most of the rate-limiting steps of an effective anti-tumor immune response in pancreatic cancer [5,6].

Pancreatic cancer bears unique immunologic hallmarks. With a low-moderate mutational burden, pancreatic cancer cells are less immunogenic and reside within a dense stromal environment. The stromal matrix, which comprised of cellular and acellular components, such as fibroblasts, myofibroblasts, pancreatic stellate cells, immune cells, blood vessels, extracellular matrix and soluble proteins such as cytokines and growth factors, contributes to tumor growth and promotes metastasis [7]. In line with the excessive desmoplasia, another conundrum of pancreatic cancer is a deficiency of vasculature, leading to impaired perfusion and drug delivery. The unique TME of pancreatic cancer gives rise to a series of challenges along multiple steps of anti-tumor immune response: a lack of strong cancer antigens or epitopes recognized by T cells (Step 1), minimal activation of cancer-specific T cells (Step 3), poor infiltration of T cells into tumors (Step 5), downregulation of the major histocompatibility complex on cancer cells (Step 6), and immunosuppressive factors and cells in the tumor microenvironment (Step 2–7). As a consequence, T cells are largely excluded from the immediate TME, which may, at least partially, explain the unresponsiveness to checkpoint blockade therapy (including anti-PD-1/PD-L1 and anti-CTLA-4) [8]. An individual pancreatic cancer patient can have a deficiency in one or multiple step(s) of anti-tumor immune response. Thus, a personalized medicine approach will ultimately be required for effective cancer immunotherapy. 

## 3. Biomarkers Identification

### 3.1. Tumor Microenvironment 

#### 3.1.1. PD-L1 Expression

The PD-1/PD-L1 pathway regulates the balance between the stimulatory and inhibitory signals needed for effective immune responses [9]. In tumors, upregulation of PD-L1 on cancer cells creates an “immune shield” to protect against immune attack from T cells and contributes to the development of T-cell exhaustion [10]. This lays the foundation of checkpoint blockade of the PD-1–PD-L pathway to unleash the effector functions of T cells and reinvigorate the killing of tumor cells, and also highlights the value of PD-L1 expression as a predictive biomarker for this class of therapy. For tumors reported with clinical response to the anti-PD-1/PD-L1 therapies including melanoma, renal cell carcinoma (RCC), non-small cell lung cancer (NSCLC) and bladder cancer, the range of PD-L1 expression falls from 14% to 100% [11,12,13]. In the KEYNOTE-010 study, the magnitude of benefit with pembrolizumab was associated with the levels of PD-L1 expression, with increased survival benefits in patients with PD-L1 expression ≥  50% of tumor cells (regardless of the staining intensity with the 22C3 clone) [14]. A further study restricting patient recruitment among PD-L1 ≥ 50% showed a significantly improved progression free survival and subsequently established pembrolizumab as a first-line treatment for metastatic NSCLC with PD-L1 expression ≥ 50% [14]. By contrast, a clinical trial based on PD-L1 selection of ≥ 5% showed no survival advantage of nivolumab as the first line treatment compared to the standard-of-care chemotherapy [15]. Of note, differences in the PD-L1 expression threshold may not be the single factor contributing to the divergent results of the above two clinical trials; other factors such as difference in assays of assessing PD-L1 expression and imbalance in baseline patient characteristics may also have contributed to the difference in the clinical trial results.

In PDACs, reports of PD-L1 expression vary from 12–90% [16,17,18,19], suggesting that we do not have a consensus on the expression of PD-L1 [20,21]. Multiple factors may have contributed to the wide range of reported expression rates including the specificity of the staining methodology, the difference in the PD-L1 positivity cutoff, the difference between primary and metastatic lesions, and the inclusion of tumor vs. immune cells in quantifying PD-L1 expression [22]. However, one important factor may be the effect of the prior treatment on the expression of PD-L1 [20,22]. Thus, extreme cautions should be taken in investigating the predictive role of PD-L1 expression for immunotherapy response in pancreatic cancer. In fact, none of the pancreatic cancer patients without mismatch repair deficiency has responded to the single-agent anti-PD-1 antibody treatment.

#### 3.1.2. Tumor Infiltrating Lymphocytes

Tumor infiltrating lymphocytes (TILs) are the immune cell context direct interacting with the TME and have been shown to act either as a predictive or prognostic factor for treatment in various cancers [21,22,23,24,25]. In a study in colon cancer, the densities of CD3^+^, CD8^+^, granulysin, and granzyme B (GZMB)^+^, and CD45RO^+^ cells in each tumor region (tumor center and invasive margin) were shown to be prognostic [23]. In melanoma, a significant correlation was observed between the presence of both TILs and B7-H1 expression in the tumor microenvironment and the response to checkpoint blockade [26]. Nevertheless, only having positive PD-L1 expression and TILs is not sufficient for pancreatic cancer responding to anti-PD-1 therapies. In our study evaluating 24 pancreatic ductal adenocarcinomas from patients who received neoadjuvant GVAX vaccination, although essentially all the tumors have induction of TILs and PD-L1 expression, the survival of patients is correlated with the infiltration of myeloid cells [27]. Therefore, only a comprehensive characterization of the tumor infiltrating immune cells would adequately support the precision medicine practice for pancreatic cancer. 

### 3.2. Biomarkers in the Genome 

#### 3.2.1. Mutations and Mutation Burden

Known as a genetic disease, cancer is initiated by mutations that activate oncogenic drivers and eventually turn normal cells into cancer cells through activation of genes that promote proliferation or suppression of genes that modulate apoptosis. In many cancers, oncogenesis is accompanied by the accumulation of mutations, which lead to generation of neoantigens that capable of eliciting potent T cell responses and drive response to current immunotherapies [4]. A growing body of evidence in the past five years have suggested that the overall mutational burden is a predictive biomarker of checkpoint blockade therapies [28,29,30,31]. For example, in tumors with higher spectrum of somatic mutational burdens, such as melanoma and non–small cell lung cancers, treatment with anti–PD-1 blockade has resulted in significantly improved survival outcome [14,32,33]. Moreover, recent research identified truncal mutations, which arise early in oncogenesis and are shared by almost all of the cancer cells, are more likely to elicit a potent anti-tumor response compared to those arise later and shared by only a subgroup of cancer cells (branch mutation) [4]. In solid tumors with mismatch repair (MMR) deficiency, higher mutational burdens were seen [34,35]. In a Phase II clinical trial of progressive metastatic carcinoma with or without MMR deficiency, whole-exome sequencing revealed a significantly increased somatic mutations per tumor (mean, 1782 vs. 73) in MMR–deficient tumors as compared with MMR–proficient tumors. This corresponds to a remarkable increase in immune-related objective response rate (40% vs. 0%) and prolonged immune-related progression-free survival rate in MMR-deficient tumors vs. MMR-proficient tumors (78% vs. 11%) [35]. In the subsequent expanded cohort which included 86 advanced MMR-deficient patients across 12 different tumor types, objective radiographic responses were observed in 53% of patients and complete responses were achieved in 21% of patients [36]. This leads to the recent approval of the microsatellite instability high condition, the phenotype of mismatch repair deficiency, as a pan-cancer biomarker for immune checkpoint blockade therapies and set a stage for the development of precision immuno-oncology. Another potential genetic biomarker is associated with point mutations affecting DNA replication—polymerase epsilon (POLE) or polymerase delta (POLD1), which have been reported to exhibit some of the highest mutational burdens identified to date and render patients with exceptionally mutated (ultramutated) cancers [37,38,39,40]. Improved survival has been observed for POLE-mutated tumors in retrospective studies of conventional therapy setting [21]; and a few case reports also reported dramatic responses to immune checkpoint blockade [41,42]. 

In pancreatic cancer, the average mutation burden was reported from 26~67 mutations per case [43,44], representing a relatively low mutational burden compared to those seen in other solid tumors. In general, PDAC with more copy number alterations (indicative of chromosomal instability) exhibited mutations in DNA break repair genes and trended toward poor prognosis [43]. Microsatellite instability (MSI) and POLE/POLD1 mutations are found in 2% and 1–2% of patients with pancreatic cancer, respectively [45]. Favorable outcomes are anecdotally reported for MMR-deficient PDAC [46], suggesting that even lower incidence of MMR deficiency would be found in the PDAC patients who require treatment. A retrospective cohort study of resected PDAC (154 in discovery cohort and 95 in replication cohort) clustered PDAC into five predominant mutational subtypes: age related, double-strand break repair, mismatch repair, and one with unknown etiology. Those with higher frequency of somatic mutations and tumor-specific neoantigens corresponded to double-strand break repair and mismatch repair subtypes, which were found to have higher expression of antitumor immunity, including activation of CD8+ T lymphocytes and overexpression of regulatory molecules (CTLA-4, PD-1, and indolamine 2,3-dioxygenase 1 [IDO1]) [47]. Moreover, a significant number of germline mutations that are associated with genes for DNA repair were found in the double-strand break repair subtype including BRCA1, BRCA2, PALB2, and ATM. These gene mutations render individuals susceptible to pancreatic cancer and are potential biomarkers for response to targeted therapy or immunotherapy. Ongoing clinical trials are testing Poly (ADP-ribose) polymerase (PARP) inhibitors in the small fraction of patients (<10%) with mutations in BRCA2 or other DNA repair genes. 

Although high mutation burden is a predictive biomarker for favorable response, individual mutations may also act as a predictor of inferior anti-cancer immune response, particularly those cancer-associated driver mutations. Mutations activating the MAP kinase pathway, for example, the KRAS and BRAF mutations, may attenuate T-cell recognition by down-regulation of the major histocompatibility complex (MHC) class I antigen-processing factors [48,49,50,51]. Notably, KRAS and BRAF is mutated in 90–95% and 3% of PDAC, respectively [43]. These alterations result in a decrease of T-cell ligands for antigen presentation (Step 2) and thus attenuating anti-tumor inflammatory response. Reduced expression of the MHC class I antigen-processing factors enables cancer cell more likely to escape from immune surveillance and attack, and poses great challenges for effective immunotherapy. Oncogenic RAS has also been shown to upregulate expression of immunomodulatory cytokines, such as IL-8 and GM-CSF [52], which subsequently induces the infiltration of myeloid derived suppressor cells (MDSC) [53]. Other mutations can also attribute to immune resistance. For example, β-2-microglobulin (B2M) and Janus kinases (JAK1 and JAK2) mutations have been reported to attribute to primary and secondary resistance to the PD-1 blockade therapy [54,55]. Truncating mutations of B2M lead to loss of surface expression of MHC class I molecules. Loss-of-function mutations of AK1/2 result in a lack of response to the interferon gamma signaling. Recognizing that B2M and JAK1/2 mutations would lead to lack of response to PD-1 blockade therapy, it has been suggested that these genes be incorporated in target gene sequencing panels to help select patients for precision cancer treatments. To validate the predictive value of genomic biomarkers with a low incidence, basket trials that incorporate precision medicine into hypothesis-driven clinical trials have emerged as a feasible solution [56]. In these clinical trials, tumors are classified based on genetic alterations instead of tumor histology. These clinical trials scale the number of patients screened for multiple genetic alterations in tumors through a large consortium or multi-institution collaboration and assign patients to a treatment arm corresponding to one particular actionable mutation. 

#### 3.2.2. Chromosomal Chaos

Aneuploidy, also known as somatic copy number alterations (SCNAs), is characterized by the presence of a chaotic chromosomal environment with abnormal number of chromosomes and chromosomal segments and has been proposed to drive tumorigenesis in various cancer types [57]. In pancreatic cancer, DNA aneuploidy is an independent factor of poor prognosis [58]. Recently, aneuploidy was found to be associated with decreased expression of cytokines responsible for tumor destruction (IFN-γ, IL-1A, IL-1B, and IL-2) [59]. In this study, compared to the mutation number, the level of SCNAs showed a stronger correlation with the cytotoxic immune signature in most of the tumor types examined, even in those whose mutation numbers positively correlated with the SCNA levels. Clinical validation of the predictive role of aneuploidy in melanoma patients treated with anti–CTLA-4 revealed that high SCNA levels were associated with a poorer response [59].

## 4. Immunotherapeutic Targets for Pancreatic Cancer

### 4.1. Immune Checkpoints 

Immune checkpoint inhibitors, including anti-CTLA4, anti-PD-1, and anti-PD-L1 antibodies, are effective as single agents in immune-sensitive cancers like melanoma, renal cell carcinoma and NSCLC, but lack efficacy in immune-quiescent or resistant cancers such as pancreatic cancer [60,61,62]. In a phase II trial, Ipilimumab was administered to 27 patients with locally advanced or metastatic pancreatic cancer. Unfortunately, no significant improvement in survival was observed [63]. As discussed previously, PD-L1 was rarely expressed on untreated pancreatic cancer. Therefore, combination strategies to leverage the potentials of other therapies to turn tumors from immunologically “cold” to “hot” are a key to achieve the response to immune checkpoint blockade. 

### 4.2. Activating or Introducing Cancer Antigen-Specific T Cells

#### 4.2.1. Tumor Vaccines

Over the last decade, great efforts have been made to explore the role of vaccines in enhancing the immunogenicity of cancer cells and boosting the T cell response in pancreatic cancer. At the center of vaccine design is the delivery of tumor antigen to antigen presenting cells (APCs), which plays a major role orchestrating the immune response [64]. Early studies with single agent tumor vaccines targeting cancer associated antigens (TSAs) showed improved immune profiles, but have been largely unsuccessful in inducing a positive clinical response [65]. On the one hand, lack of a strong immunogenicity of TSAs may have decreased the efficiency of the vaccine. On the other hand, the excessive immune suppressive TME also contributes significantly to the unresponsiveness. However, our group has raised the “Priming” hypothesis and demonstrated the role of cancer vaccine in priming the TME for immune checkpoint blockade therapies. This evidence supports the combinatorial immunotherapy strategies by providing T cells to immunologic “cold” TME followed by removing the inhibitory signals in the TME [66,67,68,69]. GVAX, which composed of two granulocyte-macrophage colony-stimulating factor–secreting allogeneic pancreatic tumor cell lines, induces T-cell immunity to cancer antigens, including mesothelin [70]. In a clinical trial of 59 patients with resectable pancreatic cancer treated preoperatively with GVAX, infiltration of T cells and development of tertiary lymphoid structures in the TME were observed in the tumors resected 2 weeks following the treatment of GVAX, suggesting that the combination therapy converted a “non-immunogenic” neoplasm into an “immunogenic” neoplasm. In addition, the vaccine therapy induces the adaptive immune resistance mechanisms, including the PD-1–PD-L1 pathway, indicates that the vaccine approach could prime pancreatic cancer for immune checkpoint and other immunomodulatory therapies [17]. This was also evidenced by another trial in which favorable objective responses were observed among metastatic PDAC patients treated with the combination of GVAX and Ipilimumab compared to ipilimumab alone [71]. Therefore, combinatorial therapy of vaccines with immunomodulatory therapies (e.g., Checkpoint blockade) or other treatment modifying TME would be the best strategy.

#### 4.2.2. Neoantigens and Neoepitopes

The recognition that neoepitope-specific T cells underlies the immune response to checkpoint inhibitors have pushed neoantigens to the wavefront. Indeed, as tumor neoantigens are non-self to the host immune system, they are less likely to induce immune tolerance or trigger autoimmunity [72]. As discussed previously, the fact that mutation/neoantigen burden drives immune response and correlates with clinical outcomes of immune checkpoint blockade strongly supports the use of neoantigens for therapeutic intervention. In a mouse model, therapeutic vaccines of synthetic mutant peptides predicted from genomics and bioinformatics approaches showed comparable outcome as checkpoint blockade in sarcomas [73]. Steven Rosenberg and his group reported that a metastatic cholangiocarcinoma patient treated with adoptive transfer of TIL containing mutation-specific polyfunctional T helper-1 cells achieved notable tumor regression. CD8^+^PD-1^+^ neoantigen-specific lymphocytes detected in the peripheral blood in melanoma patients resembled infiltrating CD8^+^PD-1^+^ cells in their tumors, implying that vaccine-induced CD8^+^PD-1^+^ lymphocytes could also traffic into the tumors [74]. These results provide evidence for the rationale to develop personalized therapies using neoantigen-reactive lymphocytes or T cell receptor (TCR) engineered T cells to treat cancer [75]. Adoptive transfer of T cells targeting the mutant KRAS as a cancer-specific neoantigen is also being tested [76].

In the initial step of developing personalized neoantigen vaccine, somatic mutations can be identified by comparing the whole exome sequences and those of matched normal cells [77,78]. Tumor RNA expression profiling will help to filter in those expressed candidate neoepitopes [79]. Subsequent prediction of putative peptides involves interrogating the class I and class II HLA binding affinities using neoantigen prediction software. The final step requires well-designed clinical study to validate the vaccine before it applied to the patients.

### 4.3. Targeting the Microenvironment

#### 4.3.1. Stromal Matrix

**Figure 2:**
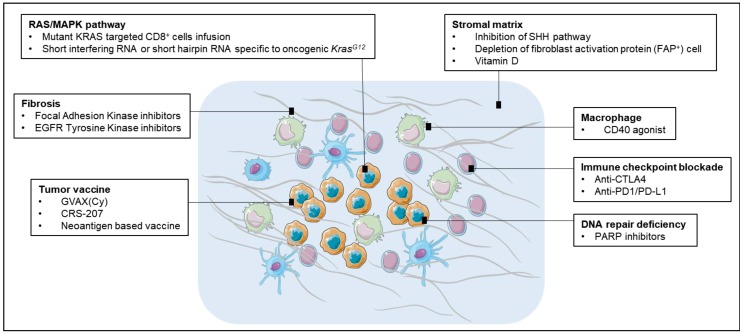
Potential immunotherapeutic targets for pancreatic cancer

Stroma cells contribute to the resistance of pancreatic cancer to chemotherapy by forming an insulating matrix around the tumor, squeezing the blood vessels and preventing chemotherapy drugs and immune cells from reaching it (Figure 2). Breaching the significant stroma barriers represents a promising strategy to improve the delivery and efficacy of systemic therapeutics agents. Various attempts have been made to target stromal profibrotic pathways, cytokines and growth factors involved in tumor desmoplasia and angiogenesis. In a mouse PDA model, inhibition of the sonic hedgehog (SHH) signaling pathway could engender a dramatic depletion of stromal components paralleled by an increase in intratumoral vascular density, leading to a significantly enhanced concentration of intracellular metabolite of gemcitabine, transient disease stabilization and a significant prolongation of survival [80]. However, tumors could develop adaptation to chronic SHH inhibition and ultimately resume stromal desmoplasia and hypovascularity [80]. Though disappointing, it provides a proof of principle that disruption of the desmoplastic stroma facilitates the delivery of therapeutic agents into PDAC. Preliminary data in mice have shown that a synthetic form of vitamin D could be used to target the protective matrix, which would increase the delivery of chemotherapy and immune cells into tumor more efficiently [81]. Another study reported depleting carcinoma-associated fibroblasts (CAFs) expressing fibroblast activation protein (FAP) resulted in the immune control of PDAC growth. The depletion of the FAP(+) stromal cell also uncovered the antitumor effects of α-CTLA-4 and α-PD-L1, indicating that the potential therapeutic value of stroma targeting agents in combination with T-cell checkpoint antagonists [8].

#### 4.3.2. CD40/CD40L Pathway

The tumor microenvironment in PDAC is predominantly immunosuppressive and exerts potent restrains for antitumor immunity. CD40 agonist antibodies (αCD40) promote APC maturation and enhance macrophage tumoricidal activity [82]. Beatty et al. reported on a small cohort of PDAC patients treated with gemcitabine chemotherapy plus anti-CD40 agonist antibodies and observed tumor regressions in a CD40-dependent mechanism by targeting tumor stroma [83]. More recent studies suggested that CD40 agonists can mediate both T-cell-independent and T-cell-dependent immune mechanisms of tumor regression in pancreatic cancer. The former mechanism involves systemic activation of macrophages that infiltrate the tumor, become tumoricidal, and facilitate the depletion of tumor stroma [84]. The latter finding suggested, when combined with chemotherapy, CD40 blockade can activate T-cell immunity and mediate major tumor regression, but this anti-tumor T-cell response is restrained by suppressive elements in the tumor microenvironment. An early clinical trial testing agonist CD40 monoclonal antibody in combination of gemcitabine was well-tolerated and associated with increased antitumor activity in patients with PDAC [85].

#### 4.3.3. RAS/MAPK Activation

With the significant role of RAS/MAPK (Mitogen-activated Protein Kinase) pathway in modifying the TME, inhibition of activated RAS downstream pathways may increase anti-tumor immune response and thus therapeutic effect of checkpoint blockade therapy. In melanoma patients, treatment with MEK (Mitogen-activated protein kinase kinase) inhibitors have shown reduced levels of immunosuppressive cytokines and increased TILs [86,87]. For triple-negative breast cancer (TNBC), the use of MEK inhibitors has enhanced the responses to immune checkpoint blockade, with an upregulation of MHC I and II on tumor cells, increase in CD8 TILs, and improved prognosis [88]. In pancreatic cancer, most efforts targeting RAS/MAPK activation pathway has been largely unsuccessful. However, simultaneously targeting RAS/MAPK pathway and providing immunotherapy hold a promise to tear down the barriers of immune suppression in the TME and increase the effectiveness of immune-based therapeutic modalities. Nevertheless, additional resistance mechanism may likely need to be identified and overcome. 

#### 4.3.4. Focal Adhesion Kinase 

Focal Adhesion Kinase (FAK), is a non-receptor tyrosine kinase that plays an critical role in cancer migration, proliferation, and survival [89], and more recently has been found to regulate pro-inflammatory pathway activation and cytokine production [90,91]. In neoplastic PDAC cells, hyperactivated FAK activity has been shown to orchestrate fibrotic and immunosuppressive TME. In the mouse model of PDAC, elevated FAK activity correlated with high levels of fibrosis and poor CD8^+^ cytotoxic T cell infiltration in tumors. However, administration of FAK inhibitors was associated with markedly reduced tumor fibrosis and decreased numbers of tumor-infiltrating immunosuppressive cells [92]. Furthermore, FAK inhibitors were shown to render previously unresponsive PDACs sensitive to chemo- and immunotherapy including anti-PD-1 and anti CTLA-4 antibodies in preclinical model of PDACs. These findings have supported the clinical testing of FAK inhibitors in combination with checkpoint inhibitors and chemotherapy for pancreatic cancer treatment.

### 4.4. Targeting DNA Repair Mechanisms

Poly (adenosine diphosphate [ADP]) ribose polymerase (PARP), which represents a powerful machinery for single-strand break repair, orchestrates the DNA damage response (DDR) and the maintenance of genomic stability inhibitors [93,94]. In murine models, PARP^−/−^ knockout mice demonstrated increased genetic instability, major DNA repair defects as well as hypersensitivity to alkylating agents and radiotherapy [95]. A synthetic lethal therapeutic effect was also proved for patients with compromised ability to repair double-strand DNA breaks by homologous recombination when treated with PARP blockade [96]. Over the past few years, PARP inhibitors (PARPi), which have been tested as monotherapies or in combination with DNA-damaging agents, have shown an efficacy against tumors with defects in DNA repair mechanisms, especially in BRCA1/2mutation–related cancers [97]. There have been great interests in the combination of immunotherapy with PARP inhibitors based on the rationale that PARPi may increase a synergic therapeutic effect. Treatment using a PARP inhibitor together with immunotherapy of CTLA-4 blockade was shown to induce long-term survival in a BRCA1-deficient ovarian tumor model, with local induction of antitumor immunity and the production of increased levels of interferon-g (IFNγ) in the peritoneal tumor environment. Several clinical trials testing the combination of PARPi and immunotherapy for PDACs are underway.

## 5. Conclusions

The advances in immunogenomics coupled with the recent fundamental advances in the understanding of immune-tumor microenvironment interaction have created opportunities for the development of more effective and personalized immunotherapy approaches for pancreatic cancer patients. Great efforts have focused on identification of biomarkers to facilitate more precise choices of immune modulatory agents. Research-driven drug discovery has led to the emergence of a variety of novel therapeutic targets that are reshaping the pancreatic cancer treatment landscape. Future directions include strategies to pool the right neoantigens/neoepitopes for personalized vaccines, to break host mechanisms of immune tolerance, to enable relevant immune cells to effectively localize to the sites of disease, and to use new drugs such as PARPi to target DNA repair deficiencies. Moreover, studies characterizing PDACs with unique neoantigens and those from long-term survivors might provide new insights into effective treatment strategies for pancreatic cancer [98]. With enormous amount of genomics and immune-biomarker data generated, more efforts should be made to the development of relational databases and bioinformatics platform to empower the practice of precision immuno-oncology. Nevertheless, before unleashing the full power of precision oncology, more studies with robust validation and well-designed clinical trials are warranted to provide evidence to support individualized immunotherapy for pancreatic cancer.
